# Myasthenia Gravis Development and Crisis Subsequent to Multiple Sclerosis

**DOI:** 10.1155/2011/291731

**Published:** 2011-05-18

**Authors:** Kurosh Gharagozli, Maziar Shojaei, Ali Amini Harandi, Nayyereh Akbari, Manouchehr Ilkhani

**Affiliations:** ^1^Loghman Hospital, Shahid Beheshti University of Medical Sciences, 13336-31151 Tehran, Iran; ^2^Department of Neurology, Loghman Hospital, Shahid Beheshti University of Medical Sciences, 13336-31151 Tehran, Iran

## Abstract

During the last decade, sporadic combination of multiple sclerosis (MS) and myasthenia gravis (MG) has been reported repeatedly. Although these are anecdotal, they are important enough to raise concerns about co-occurrence of MG and MS. Here, we present a case of an MS patient who developed an MG crisis. She had received interferon for relapsing remitting MS. Interestingly, she developed an MG crisis 4 years after the diagnosis of MS. MS and MG have relatively the same distribution for age, corresponding to the younger peak of the bimodal age distribution in MG. They also share some HLA typing characteristics. Furthermore, some evidences support the role of systemic immune dysregulation due to a genetic susceptibility that is common to these two diseases. The association may be underdiagnosed because of the possible overlap of symptoms especially bulbar manifestations in which either MG or MS can mimic each other, leading to underestimating incidence of the combination. The evidence warrants physicians, especially neurologists, to always consider the possibility of the other disease when encountering any patients either with MS or MG. Anecdotal and sporadic reports of combination of multiple sclerosis (MS) and myasthenia gravis (MG) have been raised concerns about co-occurrence of them.

## 1. Case Report

A 44-year-old woman with definite MS according to Mc-Donald criteria and a past history of hypothyroidism was followed by interferon beta-1b two times per week. Forty-three months after first MS attack, she presented with regurgitation and dysphagia that appeared 2 months ago. This was initially considered to be a new attack of MS. By history taking and neurological examination, other signs were recognized: easy fatigability (on examination), nasal speech, and ptosis. The new signs made the neurologist suspect myasthenia gravis (MG).

The diagnosis of MG was supported by positive edrophonium test. Slow repetitive nerve stimulation of spinal accessory nerve at 3 Hz shows a 23% decrement in CMAP amplitude. This amount of decrement is abnormal and is consistent with a neuromuscular junction transmission disorder, but serum acetylcholine receptor antibodies (ACh-R Ab) were negative. Other antibodies which may be present in MG patients were not assessed. Computerized tomography scan of the thorax revealed no thymic enlargement and just persistent remnants of the gland were detected. She has received 60 mg pyridostigmine bromide three times per day and interferon beta-1b was discontinued. The signs and symptoms of MG were incompletely relieved after a few days.

She was discharged with mild orofacial weakness and followed. After two weeks, she presented with severe bilateral ptosis, intermittent double vision, chocking during swallowing, and dyspnea. With diagnosis of MG crisis, the patient was admitted to intensive care unit and underwent tracheal intubation followed by plasmapheresis 4 sessions comprising exchange with albumin 2 liters in each day. In addition, prednisolone 25 mg per day was prescribed. After extubation, pyridostigmine bromide every 4 hours was administered via nasogastric tube. After plasmapheresis the patient still had diplopia and ptosis and mild dyspnea and the gag reflex was impaired. The patient had a weight of 60 Kg, as a result 120 g (2 g/kg) intravenous immunoglobulin was administered over 6 days and produced a dramatic improvement in her clinical condition. After day 8, the patient showed improvement, and, after day 14, the patient was discharged with only mild ptosis and some limitation in vertical eye movement. After 6 months, she neither developed a new attack of MS nor an MG crisis.

## 2. Discussion

Interestingly, we described for the first time crisis of MG just two months after presentation of MG symptoms. In previous years, coexistence of MS and MG was reported rarely [1]. 

Supporting evidence comes from an increased incidence of reported CNS demyelinating disease including MS in patients with MG [2]. Gotkine et al., in 2006, reported five of 214 reviewed patients with MG (2.3%) who had CNS demyelinating disease. However, only 1 patient definitely had dissemination in time and space and another possibly such as neither had typical MS. In the report by Gotkine et al., CNS demyelinating disease always occurred subsequent to the initiation of MG treatment, supporting the possibility that immunemodulating therapies including thymectomy and immunosuppression may have a role [3]. It has been suggested that immune dysregulation could be induced by thymectomy, and a considerable number of patients showed different autoantibodies in their serum years after thymectomy when compared with nonthymectomized patients and healthy ones [4]. It has been reported that the clinical course of both MG and MS is mild in most patients with this combination of neuroimmunological disorders, but the onset of MG could cause an exacerbation of MS whereas MG can be relatively unaffected by fluctuations in the clinical course of MS [1, 5]. However, we encountered a case of MS who developed MG crisis. Consequently, it seems any clinical course could be expected by these two autoimmune diseases. In the largest study in Vancouver British Colombia on patients with well-documented diagnoses of both MS and MG, 8 patients with co-occurring MS and MG were reported. The value was significantly higher than predicted by prevalence estimates. MS occurred before MG in six patients, after MG in seven patients, and within one year in two patients. According to an epidemiological survey, 15 patients have been reported in the literature with definite diagnoses of both MS and MG [1]. In a case series on 1718 patients with definite MS in Isfahan, Iran, five MG were detected (291/100,000; 0.29%) [6]. None of the six patients was positive for ACh-R Abs. Our case also was negative for acetylcholine receptor antibodies, but other series reported more positive results. As a limitation of our study, evaluation of other antibodies such as antimuscle-specific tyrosine kinase antibody, antistriational muscle antibodies, Titin, antiryanodine receptor antibodies, which are related to MG was not feasible in our setting. Occurrence of these antibodies could provide more documents regarding underlying mechanisms that are involved in combination of MG and MS. However, seronegative results for mentioned antibodies could not rule out diagnosis of MG and their assessment is not mandatory.

In addition, three Brazilian patients were reported with MG that presented distinct demyelinating diseases including two monophasic and one recurrent neuromyelitis optica, 6–18 years after the diagnosis of MG. In consistence to the previous reports, ocular and bulbar manifestation of MG were the most common findings in our case [5, 6].

Some rational mechanisms have been hypothesized for this phenomenon. First, it is a random finding that could happen by chance alone. Moreover, it has been considered as a coincidence of two autoimmune disorder [5] or MG may be triggered by interferon treatment via deviation of immune response towards a predominantly Th2 reaction [7]. It is worth to note that interferon *β*, apparently, may cause signs and symptoms of MG by itself, as has been reported in the literature [7, 8]. However, there are some reports in which MS occurred after development of MG [5].

Furthermore, two diseases have relatively same distribution for age, the younger peak of the bimodal age distribution in MG and HLA typing (A1, A2, DR3, B8) [9]. Antinuclear antibodies (ANAs) usually occur in approximately 20 to 30% of MS and 40% of MG patients. The high frequency of ANAs in MS and MG can support the role of systemic immune dysregulation [10]. Both of them are related with some autoimmune disorder. It has been suggested that there may be some genetic susceptibility that is common to this group of diseases [11]. It is of note that our patient was suffering from hypothyroidism which, in most cases, mainly in females, is due to Hashimoto's disease, another immune disorder. On the other hand, dysfunction of thyroid gland may influence the functional state of the neuromuscular transmission.

An accidental coincidence of two diseases was excluded by high significant estimation of British Colombia study as largest series in this setting. Also, the incidence of demyelinating diseases in patients with MG is much higher than expected in the general population [7].

Also, presentation of MS subsequent to MG is an argument against the triggering effect of interferon generating MG as sole etiology. Furthermore, there were some patients who developed MG during the course of MS without treatment with interferon. So, it is too simplistic to pay no heed to important combination between MS and MG. The association may be underdiagnosed because of the possible overlap of symptoms specially cranial manifestations in which either MG or MS can mimic each other manifestations leading to underestimating incidence of new ones. The evidence warrants physicians, especially neurologists, to always consider the possible occurrence of the other disease when encountering a patient with either MS or MG. This critical question should always be considered: whether the new symptom could be the first presentation of another disease or not.
